# A Critical Review of Longitudinal DNA Methylomic Changes Associated with Treatment Response in Major Depressive Disorder

**DOI:** 10.3390/ijms27136089

**Published:** 2026-07-07

**Authors:** Rosana Carvalho Silva, Danae Zareifi, Danai Giannakou, Antreas Afantitis, Alessandra Minelli

**Affiliations:** 1 Department of Molecular and Translational Medicine, University of Brescia, 25121 Brescia, Italy; rosana.carvalhosilva@unibs.it; 2 Department of ChemInformatics, NovaMechanics MIKE, 185 45 Piraeus, Greece; zareifi@novamechanics.eu (D.Z.); afantitis@novamechanics.com (A.A.); 3 Entelos Institute, Nicosia 2102, Cyprus; yiannakou@novamechanics.com; 4 Department of ChemInformatics, NovaMechanics Ltd., Nicosia 1070, Cyprus; 5 Department of Pharmacy, Frederick University, Nicosia 1036, Cyprus; 6 Genetics Unit, IRCCS Istituto Centro San Giovanni di Dio Fatebenefratelli, 25125 Brescia, Italy

**Keywords:** major depressive disorder, depression, DNA methylation, methylome, epigenomics, longitudinal epigenomic changes, treatment response, review

## Abstract

Major depressive disorder (MDD) is a prevalent psychiatric disorder in which epigenetic mechanisms, particularly DNA methylation (DNAm), may contribute to disease vulnerability and treatment response. Epigenome-wide association studies (EWAS) have increasingly investigated longitudinal methylomic changes associated with therapeutic interventions in depression; however, methodological heterogeneity limits comparability across studies. This critical review examined the methodologies and findings of longitudinal EWAS evaluating DNAm changes related to treatment response in MDD and treatment-resistant depression (TRD). A literature search identified seven studies published up to 20 June 2026. Six studies investigated non-pharmacological interventions, including electroconvulsive therapy, trauma-focused psychotherapy, and cognitive interventions, and one study explored pharmacotherapy. Considerable heterogeneity was observed regarding sample size, biospecimen type, methylation platforms, preprocessing pipelines, covariate adjustment, statistical modeling, and longitudinal sampling schedules. Most studies used Illumina EPIC array-based workflows and mixed-model analytical approaches, while one study employed sequencing-based methylation profiling. Overall, treatment-related methylation changes were modest and often limited to specific CpG sites or differentially methylated regions associated with immune, inflammatory, stress-related, and neurobiological pathways. Current evidence supports the feasibility of longitudinal EWAS approaches in depression research but highlights the need for larger cohorts, methodological standardization, and integration with multi-omics and clinical data to improve reproducibility and biomarker discovery.

## 1. Introduction

Major depressive disorder (MDD) is a highly prevalent and debilitating mental disorder characterized by symptoms such as persistent low mood, cognitive impairment, fatigue, and sleep disturbances. It represents one of the leading causes of disability worldwide, with substantial lifetime prevalence and a high burden in terms of reduced quality-adjusted life expectancy and increased comorbidity risk [1,2,3]. Despite advances in genetic research, the etiology of MDD remains only partially understood. Heritability estimates of approximately 40% suggest that factors beyond DNA sequence contribute significantly to disease risk [4], highlighting the relevance of epigenetic mechanisms in mediating environmental influences on gene expression [5].

DNA methylation, a covalent chemical modification of cytosine bases at CpG dinucleotides, is a key mechanism by which environmental exposures can produce long-lasting effects on gene regulation without altering the underlying DNA sequence. Epigenetic processes, including DNA methylation, histone modifications, and non-coding RNAs, modulate chromatin structure and DNA accessibility, influencing transcriptional activity [6]. Among these, DNA methylation is the most extensively studied and has been shown to respond dynamically to a wide range of environmental factors, including stress, early-life adversity, lifestyle factors, and pharmacological interventions [7,8]. These epigenetic modifications may contribute to interindividual variability in gene expression and to differences in vulnerability to psychiatric disorders and treatment outcomes [9,10].

In this context, epigenome-wide association studies (EWAS) approaches have been increasingly applied to investigate methylation signatures associated with depressive symptomatology, antidepressant response, and potential biomarkers of treatment resistance [11,12,13,14,15,16,17]. These studies are particularly relevant given that a substantial proportion of individuals with MDD do not respond adequately to first-line treatments, and a significant subset develops treatment-resistant depression, defined by the failure to respond to multiple therapeutic interventions [18,19]. Current treatment strategies often rely on a trial-and-error approach, which can delay effective care and increase patient burden [20]. Several studies have summarized the role of DNA methylation in treatment response in depression, including studies evaluating baseline methylation predictors and candidate-gene approaches [21]. However, little attention has been given to EWAS investigating dynamic DNA methylation changes occurring during treatment, with methylomic modifications being evaluated at multiple timepoints, not only at baseline. Longitudinal EWAS designs are valuable because they can distinguish molecular changes associated with treatment and symptom improvement from baseline epigenetic characteristics that may predict response. The identification of epigenetic biomarkers with predictive value could therefore contribute to more personalized and effective treatment selection [16,22,23].

Methodologically, EWAS typically employ high-throughput DNA methylation profiling platforms, such as the Illumina HumanMethylation450 or EPIC BeadChip arrays, combined with bisulfite conversion techniques to infer methylation status at CpG sites. Analytical pipelines generally include rigorous quality control procedures, normalization steps, and statistical models that adjust for technical variation and biological covariates, such as age, sex, cell-type composition, and population stratification. In longitudinal designs, repeated measures of DNA methylation and clinical outcomes enable the investigation of dynamic epigenetic changes over time, particularly in response to treatment [11,12,14,15,17].

This review critically examines the methodologies used to assess DNA methylation and conduct EWAS analyses in studies employing longitudinal designs in the context of depression and treatment response. It focuses on longitudinal EWAS evaluating genome-wide DNA methylation changes across treatment in MDD and TRD, with an emphasis on methodological approaches and biological findings. Specifically, it evaluates differences in experimental design, statistical modeling strategies, and covariate adjustment approaches across studies. By comparing methodological aspects, the review aims to identify strengths and limitations in the current literature and to assess how analytical choices may influence the interpretation of methylation data and the identification of clinically relevant biomarkers.

## 2. Methods

The literature search for this review was conducted using electronic databases to identify primary research articles examining longitudinal DNA methylation in relation to treatment response in MDD. Electronic searches were performed in MEDLINE/PubMed and Scopus databases using combinations of the following keywords and terms: major depressive disorder (MDD), DNA methylation (DNAm), epigenome-wide association studies (EWAS), treatment interventions, antidepressants, psychotherapy, cognitive behavior therapy, brain stimulation therapy, electroconvulsive therapy (ECT), and transcranial magnetic stimulation (TMS). The PubMed and Scopus search syntax was as follows: (“major depressive disorder” OR “depression” OR “MDD” OR “treatment-resistant depression” OR “TRD”) AND (“DNA methylation” OR “methylome” OR “methylomic” OR “epigenome” OR “epigenomic”) AND (“EWAS” OR “epigenome-wide association study” OR “genome-wide methylation”) AND (“longitudinal” OR “prospective” OR “repeated-measures”) AND (“treatment” OR “treatment interventions” OR “antidepressants” OR “psychotherapy” OR “cognitive behavior therapy” OR “cognitive intervention” OR “electroconvulsive therapy” OR “ECT” OR “transcranial magnetic stimulation” OR “TMS”). Two authors (AM, RCS) independently screened the database results to minimize selection bias and errors. The reference list of relevant studies, meta-analyses, and review articles were also manually screened to identify additional eligible publications. Studies were included if they: (a) were original peer-reviewed articles published in English; (b) included patients with MDD or TRD; (c) performed longitudinal genome-wide DNA methylation analyses before and after treatment; and (d) evaluated treatment-associated methylation changes using an EWAS approach. Studies investigating only candidate genes, cross-sectional methylation analyses, baseline predictors of treatment response without longitudinal methylation assessment, focusing on epigenetic mechanisms other than DNAm, as well as conference abstracts, review articles, editorials, book chapters, case reports, and studies based on animal models or cell cultures, were excluded. All database searches were concluded on 20 June 2026.

To improve reproducibility and comparability across studies, we extracted a structured set of methodological and quantitative reporting items from each eligible publication. This extraction framework was guided by established EWAS reporting and design recommendations and by platform-specific considerations for the Illumina MethylationEPIC array [24,25].

## 3. Results

A total of seven eligible longitudinal EWAS were included following screening and eligibility assessment. Six included studies focused on non-pharmacological interventions and one study pharmacological antidepressant treatment in patients with MDD. Three studies investigated patients with TRD undergoing ECT [12,14,15]. One study examined trauma-focused psychotherapy in TRD patients [11]. Additionally, two publications evaluated cognitive intervention therapy in patients with MDD, studying the same cohort but performing different analyses [17,26]. One study evaluated longitudinal DNA methylation changes associated with antidepressant response following 8 weeks of treatment with escitalopram or desvenlafaxine in patients with MDD [27]. The main characteristics of the included studies, including study design, sample characteristics, timepoints, treatments, examined tissues, clinical outcomes, and main findings, are summarized in Table 1. Laboratory and bioinformatics methods are summarized in Table 2, while statistical models and key EWAS findings are summarized in Table 3.

**Figure 1:**
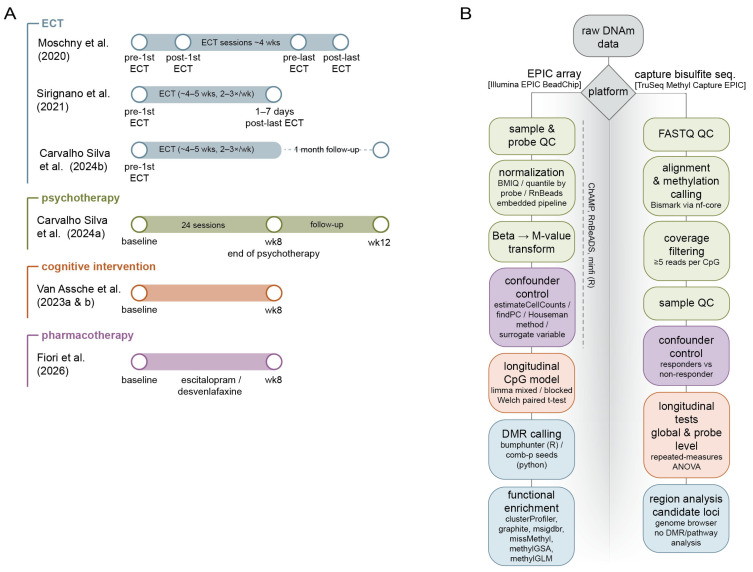
Overview of longitudinal DNAm sampling designs and bioinformatics workflows in the included studies: Moschny et al. (2020): [14]; Sirignano et al. (2021): [15]; Carvalho Silva et al. (2024b): [12], Carvalho Silva et al. (2024a): [11]; Van Assche et al. (2023a): [17]; Van Assche et al. (2023b): [26]; Fiori et al. (2026): [27]. (**A**) Schematic representation of the treatment timelines and DNAm sampling, timepoints across the longitudinal EWAS included in this review. (**B**) Generalized overview of the two main DNAm profiling and analysis workflows represented in the included studies. Colours, denote the major workflow stage: grey, input/platform; green, QC and preprocessing; purple, confounder adjustment; orange, longitudinal statistical testing; and blue, downstream region-level or functional enrichment analysis. Abbreviations: DNAm, DNA methylation; ECT, electroconvulsive therapy; QC, quality control; CpG, cytosine-phosphate-guanine site; DMR, differentially methylated region; wk, week.

To provide a visual overview of the longitudinal sampling designs and analytical heterogeneity across the included studies, Figure 1A summarizes the DNAm sampling timepoints, while Figure 1B summarized the platform-specific bioinformatics workflows represented in the review.

### 3.1. Studies’ Characteristics 

#### 3.1.1. Sample Size

The seven included EWAS presented sample sizes ranging from 12 to 34 participants in TRD cohorts and from 23 to 133 participants in MDD cohorts. Specifically, one study included 12 TRD patients [14], while others included 34 [15], 32 [12], and 30 TRD patients [11]. In MDD cohorts, sample sizes were 90 at baseline and 84 at week 8 [17] and stratified samples of 21 responders, 18 remitters, and 28 clinically stable patients [26]. In the studies performed by Van Assche and colleagues, clinical and cross-sectional EWAS analyses were conducted in a larger sample; however, the longitudinal methylomic analyses were performed in smaller subgroups of patients [17,26]. Specifically, in both studies, 73 individuals had DNA methylation available at both timepoints; however, longitudinal methylomic analyses were performed in 23 who performed better over time and were included in a within-individual paired longitudinal analysis in the first study [17]. In the second study, within-individual paired longitudinal analyses were conducted for responders (*n* = 21), remitters (*n* = 18), and stable patients (*n* = 28). In the pharmacological study performed by Fiori and colleagues, the authors included 133 patients with MDD who provided blood samples at baseline and week 8 of antidepressant treatment [27].

#### 3.1.2. EWAS Timepoints

The timepoints selected for EWAS analyses exhibited considerable variation across the included studies, with the number of assessments ranging from two to four. Although the majority of studies were based on two timepoints, the intervals between the baseline and subsequent evaluations demonstrated substantial heterogeneity. One study evaluated methylation profiles before ECT (T0—baseline) and approximately one month after the last ECT session (T1—follow-up) [12]. Similarly, blood assessments were conducted prior to the first ECT session (T1—baseline) and between one and seven days after the last ECT session (T2) [15]. In two additional studies, blood assessments were performed at baseline (T0) and eight weeks after the cognitive intervention (T1) [17,26]. Likewise, Fiori and colleagues collected blood samples at baseline (T0) and after eight weeks of antidepressant treatment (T8) [27]. One study included three timepoints, with methylation assessments conducted at baseline (T0), after eight weeks of treatment corresponding to the end of psychotherapy (T8), and four weeks after treatment during a follow-up visit (T12) [11]. Finally, one study evaluated four timepoints, with blood samples collected immediately before and 15 min after both the first and the last ECT sessions, resulting in measurements before and after the first session and before and after the final session [14]. These differences in intervention timing and DNAm sampling schedules are summarized in Figure 1A.

#### 3.1.3. Type of Treatment

Three studies investigated epigenetic changes associated with ECT in patients with TRD [12,14,15]. In one naturalistic long-term observational study, ECT was administered three times per week for up to four weeks, followed by weekly maintenance sessions; participants were later classified as responders or non-responders based on clinical outcomes [14]. Similarly, in another longitudinal observational study, patients underwent two to three ECT sessions per week, with an average of 10.82 sessions per patient and a range varying from five to 25 sessions [15]. In a further longitudinal observational study, the treatment protocol scheduled all patients for three ECT sessions per week. The mean number of administered sessions was 7.4 (±2.2 SD), with the total treatment duration determined by the clinical judgment of the attending physicians [12].

One study examined trauma-focused psychotherapy interventions. This randomized controlled trial (RCT) investigated trauma-focused psychotherapies, in which patients were randomly assigned to receive either trauma-focused cognitive behavioral therapy (TF-CBT) or Eye Movement Desensitization and Reprocessing (EMDR). Both interventions consisted of three individual 60 min sessions per week over eight weeks, in a total of 24 sessions, alongside treatment as usual [11].

Two publications using the same patient cohort examined cognitive interventions in patients with MDD [17,26]. Both studies used epigenomic data from an RCT in which participants underwent 16 treatment sessions over eight weeks, aimed at improving cognition, emotional processing, and social cognition [17,26].

The only pharmacological study investigated antidepressant treatment response in patients with MDD receiving either the selective serotonin reuptake inhibitor (SSRI) escitalopram or the serotonin-norepinephrine reuptake inhibitor (SNRI) desvenlafaxine over an 8-week treatment period. Longitudinal DNA methylation and additional gene expression changes were evaluated in relation to improvement in depressive symptoms measured using the Hamilton Depression Rating Scale (HAM-D) [27].

### 3.2. Methods of the Included Studies, Bioinformatic Analyses, and Statistical Methods

#### 3.2.1. Platforms, Biospecimens, and Laboratory Design

All seven studies utilized blood-derived DNA methylation data, but they differed in the cellular fraction analyzed and in pre-analytical sample handling. Moschny et al. uniquely profiled peripheral blood mononuclear cells (PBMCs) [14], whereas Sirignano et al. [15], Carvalho Silva et al. [11], and Carvalho Silva et al. [12] analyzed peripheral blood, and Van Assche et al. [17] and Van Assche et al. [26] analyzed whole blood. Fiori et al. also analyzed whole-blood samples collected longitudinally before and after antidepressant treatment [27].

Sample preparation procedures also varied across studies, including DNA extraction method, blood-collection conditions, DNA quantification or purification steps, storage conditions, and laboratory processing site, and some of these factors are summarized in Table 2. Pre-analytical variation may affect methylation measurements, and differences between PBMC-based and whole-blood profiles should be considered when comparing studies, since their methylation profiles are not directly equivalent.

At the platform level, six studies used the Illumina Infinium MethylationEPIC BeadChip, generating high-density array-based methylation data across approximately 850,000 CpG sites [11,12,15,17,26,27]. In contrast, Moschny et al. [14] used the TruSeq Methyl Capture EPIC Kit, representing a capture-based sequencing workflow rather than a BeadChip-based array workflow. This distinction is methodologically important because array-based studies rely on probe-intensity processing, probe filtering, normalization, and array-specific technical correction, whereas the Moschny study required sequencing-oriented processing and coverage-based methylation calling [14].

Several studies incorporated laboratory-design features intended to reduce technical variation in paired longitudinal comparisons. Carvalho Silva et al. [11], Carvalho Silva et al. [12], Van Assche et al. [17], and Van Assche et al. [26] reported sample randomization by age and sex to reduce technical bias. In both Carvalho Silva et al. studies, baseline and follow-up DNA from the same participant was placed on the same 96-well processing plate [11,12]. Similarly, Van Assche et al. [17] and Van Assche et al. [26] analyzed paired timepoints from the same participant on the same chip. In Sirignano et al. [15] arrays were processed at specialized genomic centers in Munich, supporting centralized laboratory handling for EPIC-based profiling.

#### 3.2.2. Preprocessing, Quality Control, and Normalization

Quality-control and normalization procedures were implemented in all studies, but the pipelines, filtering criteria, normalization methods, and methylation-value transformations differed across datasets (Table 2 and Table 3). Carvalho Silva et al. [11] and Carvalho Silva et al. [12] processed EPIC array data using the ChAMP package in R, excluding probes with poor detection *p*-values, low bead counts, single nucleotide polymorphism (SNP)-related ambiguity, and sex-chromosome localization. Both studies then applied Beta-Mixture Quantile normalization and transformed beta values to M-values for inferential analysis [11,12]. Sirignano et al. [15] used an in-house EPIC-array pipeline that included Illumina background correction, stringent detection filtering, call-rate-based sample exclusion, probe-type-specific quantile normalization, beta-to-M-value transformation, and removal of cross-hybridizing probes, probes with high missingness, and sex-chromosome probes. A schematic comparison of the EPIC array-based and capture bisulfite sequencing workflows is shown in Figure 1B.

Van Assche et al. [17] and Van Assche et al. [26] used the RnBeads in R for preprocessing, quality control, and differential methylation analysis, with baseline and week 8 samples preprocessed jointly before subgroup-specific analyses. Fiori et al. [27] processed EPIC-array IDAT files using minfi, applying preprocessFunnorm with noob background correction, dye normalization and correction for the first two principal components of internal control probes. Probes with poor detection, SNP overlap or sex-chromosome localization were removed, resulting in 816,087 autosomal CpGs for downstream analysis. Prior to differential methylation analysis, β-values were logit-transformed to M-values. Moschny et al. [14] followed a distinct sequencing-based workflow. Paired-end sequencing data were demultiplexed with bcl2fastq, assessed with FastQC and MultiQC, processed through the nf-core methylseq pipeline using Bismark, aligned to the hg38 reference genome, and filtered to retain methylation calls supported by at least five reads. Additional quality-control steps included HeLa-cell run controls, density-plot and dendrogram-based sample assessment, exclusion of one faulty sample, and removal of chromosomes X and Y before final statistical analysis.

#### 3.2.3. Cell-Type Composition and Technical-Confounder Adjustment

Blood cell composition and technical artifacts were addressed across the reviewed studies, although the specific adjustment strategies differed across datasets (Table 2). Carvalho Silva et al. [11] and Carvalho Silva et al. [12] estimated white blood cell fractions using the “estimateCellCounts” function from the minfi R package and included estimated granulocyte, monocyte, B cell, NK cell, CD4 positive T cell, and CD8 positive T cell estimates into their regression models. Both studies also used principal components derived from EPIC control probes to adjust for technical artifacts [11,12].

Sirignano et al. [15] estimated white blood cell fractions and included age, sex, smoking status, and control-probe principal components as covariates, omitting the granulocyte estimate from the final models because of collinearity. Moschny et al. [14] also considered leukocyte composition in downstream analyses, although the reported cell-composition and technical-confounder adjustment were less extensively specified than in the EPIC-array studies.

Van Assche et al. [17] and Van Assche et al. [26] used reference-based leukocyte deconvolution and surrogate-variable adjustment. Van Assche et al. [17] adjusted for biological sex, age, Montgomery–Åsberg Depression Rating Scale (MADRS) score, years of education, height, weight, estimated cell fractions, and surrogate variables. Cell-type fractions were estimated from whole-blood methylation data using the Houseman method [29] with the GSE110554 six-cell reference dataset [28], and technical confounding was addressed using surrogate variables both at baseline and at week 8. Van Assche et al. [26] used a similar framework, including biological sex, age, education, body weight, height, baseline MADRS, imputed missing covariates with missRanger R library, Houseman-based leukocyte estimates, and used surrogate variables to account for technical effects.

In Fiori et al. [27], no reference-based DNAm cell-type deconvolution method was reported. However, blood-cell counts were available and examined in relation to treatment response. The primary DNAm model included batch and subject effects, while a secondary model additionally controlled for sex, batch, age bins and the first four principal components.

Confounding factors and covariate-adjustment strategies varied considerably across studies, as summarized in Table 3. Commonly included covariates comprised age, sex, cell-type composition estimates, smoking status, technical principal components, and surrogate variables; however, the specific adjustment strategies differed substantially between studies. Medications were generally maintained relatively constant throughout the study periods and were not explicitly incorporated into the statistical models, with the exception of the pharmacological study by Fiori et al. [27].

Overall, cell-composition adjustment differed substantially across studies. Moschny et al. [14] analyzed isolated PBMCs rather than whole blood, which reduces some whole-blood cellular heterogeneity but introduces potential variation related to cell isolation and immune-cell subset recovery. Sirignano et al. [15] estimated white blood cell fractions and included five estimated fractions in the model, excluding the granulocyte estimate because of collinearity. In Carvalho Silva et al. [11] and Carvalho et al. [12], the authors used the minfi estimateCellCounts to estimate granulocytes, monocytes, B cells, NK cells, CD4+ T cells and CD8+ T cells [11,12]. Van Assche et al. [17,26] applied the Houseman method using the GSE110554 six-cell reference panel optimized for EPIC-array whole-blood deconvolution [28,29]. These differences in tissue fraction, deconvolution reference, and covariate inclusion are summarized in Table 2 and Table 3.

#### 3.2.4. Statistical Modeling of Longitudinal Methylation Changes

Most studies adopted an EWAS framework, but longitudinal methylation change was modeled using different statistical approaches and clinical endpoint definitions (Table 1 and Table 2). Carvalho Silva et al. [11] and Carvalho Silva et al. [12] used mixed linear models implemented in limma, with patient identifier included as a blocking factor. Differentially methylated probes were primarily interpreted using suggestive site-level thresholds, whereas region-level significance was evaluated separately through permutation-based differential methylated region (DMR) analyses. In Carvalho Silva et al. [11], these models evaluated methylation changes across psychotherapy timepoints and in relation to psychotherapy modality, symptom variation, treatment response, and relapse-related outcomes. In Carvalho Silva et al. [12], the same general framework was applied to TRD patients undergoing ECT to assess time effects, MADRS-based symptom variation, response status, and sex-stratified effects.

Sirignano et al. [15] also relied on limma-based mixed linear models, with participant ID included as a blocking factor and methylation M-values used for downstream association testing. The main effects of interest were response status, timepoint, and the interaction between timepoint and response. Response was modeled both as a binary endpoint, defined by a greater than 50% reduction in HDRS score, and as a continuous symptom-change variable (ΔHDRS), allowing categorical and dimensional ECT outcomes to be evaluated within the same modeling framework. Site-level significance was evaluated using false-discovery correction, with additional suggestive CpG-level results reported below genome-wide significance [15].

Moschny et al. [14] used a distinct repeated-measures ANOVA framework. The authors first tested longitudinal changes in global DNA methylation using repeated-measures models stratified by response status and then applied probe-level repeated-measures ANOVA to examine time, response-group, and time-by-response effects. Only samples with complete methylation data across all four timepoints were included in the final methylation analyses. Probe-level results were evaluated using FDR correction, combined with a minimum methylation-variance criterion for candidate selection [14].

Van Assche et al. [17] and Van Assche et al. [26] combined cross-sectional and within-individual analytical strategies. Van Assche et al. [17] used limma within the RnBeads framework to compare cognitively impaired and non-impaired participants at baseline and at week 8, followed by Welch’s *t*-test for paired longitudinal analysis among participants who improved cognitively over time. Van Assche et al. [26] similarly used limma for cross-sectional response versus non-response contrasts and Welch’s *t*-test for within-individual analyses of response, remission, and stable clinical course over the 8-week intervention period. The latter study also compared overlap among the top-ranked CpGs across analyses as an exploratory strategy to identify dynamic early treatment-related methylation signatures. Both Van Assche et al. analyses used genome-wide CpG-level thresholds for primary EWAS interpretation, while additional longitudinal findings were discussed as top-ranked or overlapping CpG signals rather than as genome-wide significant replicated loci.

Fiori et al. [27] modeled the relationship between change in methylation from baseline to week 8 and antidepressant response, defined using the HAM-D T8/T0 ratio, with limma. Batch and subject were included as fixed effects in the primary model. A secondary limma mixed-effects model used subject as a blocking factor through duplicateCorrelation and included sex, batch, age bins and the first four principal components. In addition, RNA-seq data were analyzed using DESeq2, and methylation-expression relationships were assessed using Spearman correlations.

#### 3.2.5. Region-Based and Pathway-Level Analyses

Several of the studies complemented single-CpG EWAS analyses with region-based or pathway-level approaches, thereby broadening the interpretive framework beyond isolated CpG findings. Carvalho Silva et al. [11] used the bumphunter R package for DMR analysis, requiring clusters of at least seven probes within 300 base pairs and estimating significance through 250 permutations. Carvalho Silva et al. [12] used a similar bumphunter-based approach, but required at least eight probes per cluster and likewise applied permutation-derived regional significance estimates. Sirignano et al. [15] used the comb-p package for DMR analysis, with a seed *p*-value threshold of 0.001 and a maximum inter-probe distance of 500 base pairs. These differences in DMR algorithms, clustering parameters, enrichment tools, gene-set resources, and correction thresholds are summarized in Table 3.

To complement this methodological overview, we extracted quantitative evidence (Table 1, Table 2 and Table 3) to support reporting completeness, reproducibility, and cross-study comparability. Together, Table 1, Table 2 and Table 3 summarize the clinical design, treatment modality, longitudinal sampling, sample size and attrition, DNAm platform and coverage, genome build, CpGs passing QC, normalization, cell-deconvolution strategy, statistical model, covariates, multiple-testing correction, significant differentially methylated probes (DMPs)/differentially methylated regions (DMRs), direction and effect-size reporting, FDR/q-values, and pathway analysis across studies.

Functional enrichment and annotation strategies also varied across studies. Carvalho Silva et al. [11] and Carvalho Silva et al. [12] performed clusterProfiler-based enrichment analyses, incorporating gene-set resources such as graphite and msigdbr. Fiori et al. [27] did not report a DMR-based analysis, but performed Gene Ontology enrichment using clusterProfiler on the top 0.1% of probesets annotated to genes. Sirignano et al. [15] used missMethyl for Gene Ontology enrichment analysis, whereas Van Assche et al. [26] used methylGSA for methylation-informed pathway testing. Van Assche et al. [17] relied more on overlap-based interpretation of prioritized CpGs and external genomic annotation resources, including the UCSC Genome Browser, Alliance of Genome Resources, and GeneCards, to contextualize findings related to cognitive improvement. Moschny et al. [14] did not report a directly comparable array-style DMR or pathway-enrichment workflow, and the analysis remained primarily focused on global methylation, probe-level effects, and candidate loci.

#### 3.2.6. Methodological Heterogeneity and Comparability

Taken together, the included studies are comparable at the level of broad longitudinal EWAS design, but not at the level of uniform locus-level inference. The main sources of heterogeneity include differences in biological material, methylation platform, sampling schedule, preprocessing pipeline, covariate adjustment, statistical modeling, DMR detection, enrichment analysis, and significance thresholds (Table 2 and Table 3). This is particularly important because Moschny et al. [14] used PBMC-based methyl-capture sequencing, whereas Sirignano et al. (2021) [15], Carvalho Silva et al. [11], Carvalho Silva et al. [12], Van Assche et al. [17] and Van Assche et al. [26] used EPIC array-based workflows.

Multiplicity correction and reporting thresholds also varied across studies, including FDR- or q-value-based CpG-level correction, suggestive site-level thresholds, permutation-derived DMR significance, Šídák correction for regional analyses, and nominal or adjusted pathway-level thresholds.

These methodological differences limit direct comparison of individual CpGs or DMRs across studies. Differences in array versus sequencing workflows, cell-composition adjustment, longitudinal modeling strategy, regional clustering criteria, pathway-enrichment tools, and correction thresholds can all influence which methylation signals are detected and how they are prioritized. Accordingly, the review should emphasize shared analytical patterns and recurring biological themes, while treating direct CpG- or DMR-level overlap across studies with caution.

Reporting completeness also varied across the included studies. Moschny et al. [14], Sirignano et al. [15], Carvalho Silva et al. [11], and Carvalho Silva et al. [12] reported several key analytical parameters, including filtering thresholds, genome build or annotation framework, covariate structure, and DMR or probe-level criteria. In the studies by Van Assche et al. [17] and Van Assche et al. [26], the main analytical framework was reported, including RnBeads, covariates, cell-type deconvolution, surrogate variables, limma, Welch’s tests, and pathway analysis, but some lower-level quality control (QC) or preprocessing thresholds were less explicit in the main text. Given the small sample sizes and frequent reliance on suggestive, region-based, or pathway-level findings, transparent reporting of software versions, QC thresholds, annotation resources, and full result tables remain essential for improving reproducibility in future longitudinal EWAS.

Importantly, none of the included studies reported replication in an independent external cohort, and several authors described their findings as exploratory or hypothesis-generating, emphasizing the preliminary nature of the reported methylation signals.

### 3.3. Main Findings of the Included Studies

In the first ECT study, the longitudinal analyses did not reveal significant global methylation changes across time or between responders and non-responders. However, probe-level analyses identified specific CpG sites showing dynamic methylation changes during treatment, including loci within *AQP10* and *TRERF1*, suggesting that although global methylation remains stable, localized methylation changes may be associated with ECT response [14].

In the second study evaluating ECT effects, longitudinal analyses identified a CpG site within the *TNKS* gene significantly associated with categorical treatment response. In addition, suggestive longitudinal methylation changes were observed in genes such as *FKBP5*, and region-based analyses identified differentially methylated regions associated with symptom improvement. These findings indicate that ECT is associated with specific methylomic alterations linked to stress-related and neurobiological pathways involved in treatment response [15].

In the third ECT study, the longitudinal EWAS identified nominally significant differentially methylated positions and regions in genes implicated in psychiatric and immune-related processes, including *ADARB1* and *SLC25A24*. Importantly, sex-stratified analyses revealed significant differentially methylated regions in female patients, enriched for pathways related to trauma and immune function. These findings are consistent with the possibility that ECT is associated with longer-term and sex-specific epigenomic modifications associated with treatment response [12].

In the context of trauma-focused psychotherapy, longitudinal analyses revealed that psychotherapy was associated with several methylomic changes, including 110 differentially methylated regions enriched in inflammation-related pathways, particularly those involving TNF signaling. When stratified by intervention type, EMDR was associated with a greater number of significant regions (141 DMRs), also enriched in inflammatory processes. These results suggest that trauma-focused psychotherapy was associated with longitudinal methylomic changes related to immune and inflammatory regulation, potentially reflecting biological processes associated with therapeutic response [11].

In studies examining cognitive intervention, longitudinal within-individual analyses did not identify genome-wide significant CpGs but revealed subthreshold signals and overlap across analyses. In individuals improving over time, no CpGs reached genome-wide significance, although overlap with 8-week findings was observed, with the most notable CpG located in the *EBF3* gene. A total of 65 CpG sites were linked to cognitive improvement over time, indicating that subtle methylomic changes may be linked to cognitive response to intervention [17].

Finally, in another study conducted by the same group, within-individual longitudinal analyses identified 13 CpGs associated with treatment response and 11 CpGs associated with remission, although none reached genome-wide significance. In responders, the top signal was located near *SOX4*, with evidence of hypermethylation over time and enrichment in sodium transport pathways. In remitters, one CpG near genome-wide significance was identified in the promoter region of *LIN37*, with pathway analyses highlighting phosphatase regulation and synaptic functioning. In the stable group, 14 CpGs showed suggestive significance, with no overlap with CpGs identified in the response or remission analyses. However, partial overlap emerged at a broader level, suggesting shared longitudinal signatures and several CpGs appeared to be specifically linked to depression recovery processes [26].

The only pharmacological longitudinal EWAS identified one epigenome-wide significant CpG site within *SCN7A* whose methylation change over the 8-week treatment period was associated with HAM-D symptom change. A second highly ranked CpG, cg17944171 annotated to *IQANK1*/*FAM83H-AS1*, showed nominal evidence. Additional response-associated CpGs were observed in genes including *ATP11A*, *CAMSAP1*, *NSD1*, *VPS13B*, and *ABCB10*. Integration with RNA-sequencing data identified coordinated methylation-expression changes in 10 genes, including *MGAT5* and *RUNX1*. Gene ontology analyses highlighted pathways related to small GTPase-mediated signaling and Wnt signaling, suggesting that dynamic epigenetic regulation of neurodevelopmental and signaling pathways might be relevant to antidepressant response. These findings support the relevance of longitudinal methylation-expression integration in pharmacological antidepressant-response studies, while remaining hypothesis-generating and requiring replication [27].

## 4. Discussion

This review represents, to our knowledge, the first comprehensive synthesis of the main findings and methodologies used in longitudinal EWAS of depression in relation to treatment response. It differs from previous reviews of DNA methylation and treatment response in depression because it specifically focuses on longitudinal epigenome-wide association studies examining genome-wide methylation changes across treatment. Previous reviews, including Dahrendorff et al. [21], primarily synthesized candidate-gene studies, cross-sectional analyses, and baseline methylation predictors of treatment response. In contrast, the present review concentrates on dynamic methylomic changes occurring during treatment and provides a detailed comparison of EWAS methodologies, preprocessing workflows, statistical models, DMR analyses, pathway analyses, and longitudinal study designs.

Notably, only seven studies met the inclusion criteria for longitudinal EWAS investigations, with two publications referring to the same patient cohort but performing distinct analyses [17,26]. Six included studies evaluated non-pharmacological interventions, including ECT, trauma-focused psychotherapy, and cognitive-based approaches. Only one study investigated longitudinal methylomic changes associated with antidepressant treatment response in patients with MDD [27]. This is remarkable given that pharmacological treatments remain the most widely used therapeutic approach for MDD [30,31]. Two studies included in this review were conducted by members of the present review team [11,12]. To minimize potential bias, study selection was revised independently by two reviewers according to eligibility criteria, and all studies were evaluated using the same methodological strategy. The included studies were characterized by relatively small sample sizes, ranging from 12 to 133 participants in the longitudinal EWAS analyses. Fiori et al. [27] included 133 patients with MDD, representing the largest cohort among the reviewed longitudinal EWAS. In the studies performed by Van Assche and colleagues, cross-sectional EWAS analyses were conducted in larger samples (*n* = 73); however, longitudinal methylomic analyses were performed in smaller subgroups of patients classified according to response, remission, and clinical stability over time [17,26]. These small sample sizes reduce statistical power and limit the ability to detect the small effect sizes typical of epigenome-wide studies. None of the studies included reported formal a priori power calculations, making it difficult to assess whether the sample sizes were adequate to detect the small effect sizes typically observed in EWAS. Despite this limitation, the included studies reported several corrected, suggestive, region-level, or subgroup-specific methylation signals, particularly when more homogeneous clinical groups were considered. Indeed, when more homogeneous groups are selected, such as patients with trauma exposure or those with TRD [11,12], or subgroups classified as responders and remitters [26], significant findings tend to emerge more consistently despite limited statistical power. This suggests that reducing clinical heterogeneity may be associated with an increased likelihood of detecting epigenetic signals, even in smaller cohorts.

Overall, the reviewed studies show that longitudinal EWAS in depression treatment research follow a broadly consistent analytical logic, but they are highly heterogeneous in how that logic is actually implemented. Most used Illumina EPIC array-based workflows with R-based tools [11,12,14,15,17,26] whereas Moschny et al. [14] employed a capture-based bisulfite sequencing workflow processed through different pipelines. Fiori et al. [27] also used an EPIC array-based approach but incorporated additional integration with longitudinal RNA-sequencing data, extending the analytical strategy beyond methylation-only analyses. These differences affect genomic coverage, filtering, normalization, methylation quantification, DMP and DMR detection, and downstream biological interpretation. Because of this, the limited overlap of CpG-level findings across studies should not be viewed purely as biological inconsistency, as it also reflects differences in platform choice, biospecimen types, preprocessing strategy, covariate adjustments, statistical modeling, regional analysis, and multiple-testing thresholds.

A genuine strength of the included studies is that most attempted to account for the longitudinal or paired structure of their data, using mixed or blocked linear models, repeated-measures ANOVA, or within-individual paired tests. Even so, differences in model specification, covariate adjustment, and significance thresholds limit direct comparability across studies. Limma-based models with patient blocking and cell-type composition adjustment offer a robust framework for array-based EWAS, but they are not directly comparable with repeated-measures ANOVA or Welch’s paired tests used in other studies.

The same applies to DMR and pathway analyses. While these approaches help move beyond single-CpG findings, they were implemented using different algorithms, annotation strategies, gene-set databases, and significance thresholds in small longitudinal cohorts; these analytical choices can meaningfully influence which CpGs, DMRs, and pathways are prioritized. From a bioinformatics perspective, the current literature does support the feasibility of longitudinal methylome profiling in treated MDD and TRD cohorts, but does not yet provide a harmonized analytical basis for robust cross-study biomarker replication.

Regarding the results, the findings suggest that treatment-related DNA methylation changes in MDD are usually subtle, often not reaching genome-wide significance, and are more frequently observed at the level of specific CpG sites or differentially methylated regions. Across ECT studies, no consistent global methylation changes were observed. However, probe- and region-level analyses identified candidate loci associated with treatment response, including genes involved in stress regulation, immune function, and neurobiological processes [12,14,15]. Psychotherapy and cognitive intervention studies identified longitudinal methylation changes enriched in inflammatory and synaptic pathways, suggesting that treatment response may be associated with dynamic epigenetic modulation of biological systems related to neuroplasticity and immune regulation [11,17,26]. In contrast, the pharmacological study by Fiori et al. [27] identified one epigenome-wide significant CpG within SCN7A associated with antidepressant response, together with additional response-associated loci and coordinated methylation-expression changes involving genes such as MGAT5 and RUNX1. Pathway analyses highlighted Wnt signaling, a pathway previously implicated in depression and antidepressant response, suggesting that longitudinal pharmacological treatment may be associated with measurable epigenomic and transcriptomic adaptations linked to clinical improvement. However, because Fiori et al. [27] remains the only pharmacological longitudinal EWAS identified in this review, its findings should be interpreted as an important but preliminary extension of the field rather than as replicated pharmacological-response biomarkers. These findings are consistent with the multifactorial and heterogeneous nature of MDD and its treatment response. It should be noted that all included studies relied on blood-derived DNA methylation, which may not fully reflect methylation dynamics in brain tissue or treatment-related molecular changes within specific neural cell populations. Although peripheral methylation can capture systemic immune, inflammatory, and stress-related processes relevant to MDD, caution is required when inferring central neurobiological mechanisms from blood-based EWAS findings. Indeed, cross-tissue studies indicate that blood methylation may be informative for selected loci but does not generally reflect methylation patterns in brain tissue [32,33].

A related limitation is residual confounding from blood-cell composition and other blood-based EWAS covariates. Inadequate control for cellular heterogeneity can inflate false-positive findings in blood-based EWAS [34]. Although most included studies attempted some form of cell-composition adjustment, the reference panels, estimated cell types, and covariates differed across studies, and PBMC-based and whole-blood analyses are not directly interchangeable. Reference-based deconvolution also fails to resolve finer immune-cell subsets, activation states, or treatment-related changes in the immune composition. Other potential confounders relevant to psychiatric treatment cohorts, including smoking, BMI, psychotropic and immunomodulatory medication, circadian timing of blood collection, sleep disruption, and recent stress exposure, were not uniformly measured or adjusted across studies. Such factors should be taken into account when interpreting the modest and largely non-replicated DMP and DMR signals.

The extraction of study-level evidence indicates that the current longitudinal EWAS literature in depression is more suitable for identifying recurring patterns than for estimating reproducible methylation effect sizes. Across the included studies, corrected genome-wide CpG-level findings were rare, and many reported signals were nominal, region-based, pathway-level, or derived from subgroup analyses. Effect sizes were inconsistently reported, with several studies identifying candidate DMPs or DMRs without providing locus-level Δβ, M-value change, confidence intervals, or complete adjusted significance values. This limits the ability to compare the magnitude of methylation change across interventions, even when the implicated genes or pathways appear biologically plausible. Consequently, the strongest synthesis supported by the available evidence is not a replicated set of treatment-response CpGs, but a recurring involvement of biological processes across heterogeneous treatment contexts. These findings should therefore be interpreted as hypothesis-generating rather than as validated epigenetic biomarkers of treatment response.

Beyond these analytical differences, heterogeneity was also evident in the clinical design of the included studies, including treatment modality, sampling schedule, tissue source, and outcome definition. Differences were evident in study design (number and timing of EWAS timepoints), treatment modalities (ECT, psychotherapy, cognitive intervention, and pharmacological antidepressant treatment), tissue types (peripheral blood, PBMCs, whole blood), bioinformatics and statistical methods. With respect to treatment type, the diversity of interventions limits direct comparability but provides a broader perspective on both shared and treatment-specific epigenetic mechanisms. Such heterogeneity has also been highlighted in previous reviews of epigenetic studies in depression and remains a major challenge for the field, contributing to variability in findings and limiting reproducibility across studies [21]. Longitudinal epigenomic studies focusing on treatment response in depression remain scarce. Although several investigations have explored DNA methylation in relation to treatment response in MDD, most have relied on cross-sectional designs or on isolated pre- or post-treatment measurements, without investigating longitudinal changes throughout treatment [13,16,21,23,35,36]. Longitudinal methylomic approaches are particularly valuable for distinguishing early from later epigenomic changes associated with treatment response. Future research should include larger, well-powered longitudinal EWAS to better understand the temporal dynamics of DNA methylation during treatment. Integrating DNA methylation data with other omics layers, such as transcriptomics and genomics, as well as clinical and environmental data, may provide a more comprehensive understanding of treatment response. The multi-omics strategy employed by Fiori et al. [27], combining longitudinal DNA methylation and transcriptomic profiling, represents a promising direction for future studies aiming to identify biologically meaningful treatment-response signatures. The use of standardized methodologies, including consistent covariate adjustment and analytical pipelines, would also improve comparability across studies. Future studies should expand to include pharmacological interventions, as well as more diverse populations, to improve generalizability and clinical applicability. Although the present review identified one pharmacological longitudinal EWAS, additional investigations across different antidepressant classes and treatment settings remain necessary.

Furthermore, the methodological heterogeneity across included studies, including differences in DNA methylation platform, tissue or cell fraction, normalization procedure, cell-type deconvolution approach, genome build, statistical model, DMR algorithm, pathway-analysis method, and multiple-testing threshold, limits direct comparability and precludes formal meta-analytic synthesis. This reinforces the need for standardized EWAS reporting frameworks and platform-specific reporting of EPIC-array quality-control procedures [24,25,37].

In conclusion, longitudinal EWAS investigating treatment-associated DNA methylation changes in depression remain relatively scarce. Current evidence suggests that methylation changes associated with treatment courses can be detected; however, findings are generally subtle, heterogeneous, and insufficiently replicated across independent cohorts. Differences in tissue source, analytical pipelines, covariate adjustment, statistical modeling, and outcome definitions remain barriers to reproducibility. Future studies should incorporate larger longitudinal cohorts, standardized EWAS methodologies, replication samples, pharmacological and non-pharmacological interventions, and integrated multi-omics approaches. Indeed, many reported methylation signals should be regarded as hypothesis-generating associations with treatment courses or symptom changes rather than evidence of causal biological mechanisms. Addressing current limitations will be essential for advancing epigenomic biomarkers and supporting the development of personalized therapeutic strategies for depression.

## Figures and Tables

**Table 1:** The table summarizes the main clinical characteristics, study designs, and main results from the included studies.

Authors	Longitudinal EWAS Sample Size	Sample Attrition	EWAS Timepoints/*n* per Timepoint	Treatment and Intervention Assignment	Tissue Examined	Clinical Outcomes/Response or Remission Definitions	Main Results
[14]	*n* = 12 (TRD patients)	Initial cohort *n* = 17; final longitudinal EWAS *n* = 12; attrition/exclusion = 5/17, 29.4%, mainly due to incomplete DNAm data across all four timepoints and QC-related exclusion	4 timepoints: 1: before the first ECT, 2: after the first ECT, 3: before the last ECT, 4: after the last ECT *n* = 12 in every timepoint	ECT Treatment assignment based on clinical indication; no randomization	PBMCs	Response definition: ≥50% reduction in MADRS score after treatment	No global methylation differences observed across timepoints or between ECT responders (*n* = 8) and non-responders (*n* = 4). EWAS identified 13 significant CpGs sites across 10 loci.
[15]	*n* = 34 (TRD patients)	No reported attrition	2 timepoints: baseline, prior to the first ECT session (T1) and 1 to 7 days after the last ECT session (T2) *n* = 34 in every timepoint	ECT Treatment assignment based on clinical indication; no randomization	Peripheral blood	Binary response defined as >50% HDRS decrease; continuous response modeled as ΔHDRS	25 patients were responders and 9 were non-responders. One CpG in *TNKS* was significantly associated with binary ECT response. Top continuous-response signal included *FKBP5*, related to stress disorders.
[12]	*n* = 32 (TRD patients)	No reported attrition	2 timepoints: baseline (before ECT treatment, T0) and 1 month after the last ECT session (T1) *n* = 32 in every timepoint	ECT Treatment assignment based on clinical indication; no randomization	Peripheral blood	Response defined as >50% MADRS decrease at T1; symptom improvement also assessed as percentage MADRS variation from baseline	23 patients were responders and 9 were non-responders. Longitudinal analyses found nominal DMPs/DMRs in psychiatric and immune-related genes; sex-stratified analyses (*n* = 22) showed significant DMRs enriched in trauma- and immune-related pathways in females.
[11]	*n* = 30 (TRD patients)	30 enrolled/profiled at T0 and T8 → 27 with T12 methylation data; 3/30 lost for T12 methylation follow-up, all from the EMDR group; attrition = 10%. No samples were excluded during QC because no sample had >10% missing/NA values	3 timepoints: baseline (T0, baseline) 8 weeks (T8, end of treatment), 12 weeks (T12, follow-up) *n* = 30 at T0 and T8 *n* = 27 at T12	Trauma-focused psychotherapy: TF-CBT (*n* = 12) and EMDR (*n* = 18) Patients assigned blinded to TF-CBT or EMDR	Peripheral blood	Response defined as >50% reduction in MADRS at T12. Symptom improvement defined as the % variation (Δ) of MADRS score compared to baseline	Longitudinal analysis showed that psychotherapy was associated with 110 DMRs enriched for inflammation-related genes and TNF signaling; EMDR showed 141 significant regions with similar inflammatory enrichment.
[17]	*n* = 23 (MDD patients): within-individual paired longitudinal cognitive-improvement analysis	Original RCT sample *n* = 112 → 3 excluded based on ancestry → DNA methylation data available for *n* = 101 → baseline EWAS *n* = 90 and week-8 EWAS *n* = 84 after QC/phenotype matching → paired DNAm *n* = 73 → longitudinal improver analysis *n* = 23. This reflects both sample attrition and analytic subset restriction	2 timepoints: baseline (T0) and 8 weeks after the intervention (T1) *n* = 23 in every timepoint	Cognitive intervention	Whole blood	Cognitive impairment is defined as impairment in at least one objective THINC-it task, using task scores ≥ 1 SD below population norms. Cognitive improvement defined as improvement in objective THINC-it impairment status over 8 weeks, e.g., fewer impaired tasks at week 8 than baseline	No genome-wide significant CpGs were found for cognitive impairment; top signals involved regulatory regions including *NTRK2*, with 65 longitudinal CpGs linked to cognitive improvement.
[26]	*n* = 21 (MDD patients with response), *n* = 18 (MDD patients presenting remission), *n* = 28 (MDD patients with clinical stability)	Original RCT sample *n* = 112 → 3 excluded based on ancestry → DNAm available for *n* = 101 → baseline analytic *n* = 90 → week-8 analytic *n* = 84 → paired DNAm *n* = 73. Response (*n* = 21)/remission (*n* = 18)/stable (*n* = 28) analyses were further restricted to phenotype-defined subgroups	2 timepoints: baseline (T0) and 8 weeks after the intervention (T1) *n* = 21 (MDD patients with response), *n* = 18 (MDD patients presenting remission), *n* = 28 (MDD patients with clinical stability) in every timepoint	Cognitive intervention	Whole blood	Response defined as ≥50% reduction in MADRS score from baseline to week 8. Remission defined as MADRS ≤ 9 at week 8 with baseline MADRS > 9. Stable course defined as no MADRS change > 5 points in either direction	-Within-individual response analysis: 13 CpGs associated with response, small increases in methylation and enrichment in sodium transport pathways. -Within-individual remission analysis: 11 CpGs linked to remission, with increased methylation in pathways related to synaptic function and phosphatase regulation. -Within-individual analysis of the stable group: 14 CpGs identified with no overlap with response or remission.
[27]	*n* = 133 MDD patients	Total treatment cohort *n* = 154 → patients with longitudinal DNA methylation data *n* = 133. RNA-seq data available for *n* = 141 and blood-cell counts for *n* = 136. Reasons for missing DNAm/RNA data were not explicitly reported.	2 timepoints: baseline (T0) and 8 weeks (T8) *n* = 133 in every timepoint	Pharmacotherapy: randomized treatment with escitalopram (*n* = 73, 10 mg, increased to 20 mg if needed) or desvenlafaxine (m = 81, 50 mg, increased to 100 mg if needed)	Peripheral blood	Depressive symptoms assessed at T0 and T8 using Hamilton Depression Rating Scale (HAM-D). Continuous measure of response assessed with the ratio of HAM-D at T8 over T0. Response defined as any change in depressive symptoms over time, regardless of scale or direction.	Two genomic loci, located in the sodium voltage-gated channel alpha subunit 7 and IQ motif and ankyrin repeat containing 1 gene, showed methylation changes associated with symptom changes. Gene ontology analysis indicated that these methylation changes were enriched in biological pathways, including small GTPase and Wnt signaling. Methylation changes in 10 genes were associated with improvements in depressive symptoms, with corresponding gene expression levels at week 8 correlating with symptom severity.

Abbreviations: CpG, cytosine-phosphate-guanine dinucleotide; DMP, differentially methylated probes; DMR, differentially methylated regions; ECT, electroconvulsive therapy; EMDR, Eye Movement Desensitization and Reprocessing; EWAS, epigenome-wide association studies; HAM-D/HDRS, Hamilton Depression Rating Scale; MADRS: Montgomery–Åsberg Depression Rating Scale; MDD, major depressive disorder; NA, non-available; *NTRK2*, Neurotrophic Tyrosine Kinase Receptor Type 2; PBMCs, peripheral blood mononuclear cells; QC, quality control; RCT, randomized controlled trial; TF-CBT, trauma-focused cognitive behavior therapy; TNF, tumor necrosis factor; TRD, treatment-resistant depression.

**Table 2:** Laboratory and bioinformatics methodology in longitudinal EWAS of treatment response in MDD.

Authors	Platform	CpG Coverage	Genome Build	CpGs Passing QC	Normalization Method	Cell Deconvolution
[14]	TruSeq-Methyl Capture EPIC targeted methyl-seq (Illumina, Inc., San Diego, CA, USA); sequenced on Illumina NextSeq 550 (Illumina, Inc., San Diego, CA, USA)	Targeted capture assay designed to cover > 3.3 million CpGs	hg38 Ensembl reference genome without decoy sequences	1,476,812 CpG sites after probe design QC; methylation calls required ≥ 5 reads; X and Y chromosomes excluded	No separate normalization method; data processed using bcl2fastq, FASTQC/MultiQC, nf-core methylseq and Bismark. HeLa DNA used as technical QC across sequencing runs.	PBMCs isolated, but no cell-type deconvolution or cell-composition adjustment described.
[15]	Illumina Infinium MethylationEPIC array (Illumina, Inc., San Diego, CA, USA)	>850,000 CpG sites assayed	Not reported; CpG chromosome/base-pair positions reported, and candidate-gene annotations used UCSC Genome Browser NCBI curated RefSeq retrieved 10 August 2018	Not reported; probes filtered for detection *p*-value, sample call rate (<95% exclusion), cross-hybridization, missing rate > 0.02, and X/Y chromosome location	Illumina background correction; quantile normalization separately for six probe types; beta values converted to M-values for downstream analysis	White blood cell fractions estimated using the Houseman method; five of six estimated cell fractions included as covariates; granulocyte estimate omitted due to collinearity/high VIF
[12]	Illumina Infinium MethylationEPIC 850K BeadChip array (Illumina, Inc., San Diego, CA, USA); scanned using Illumina HiScan (Illumina, Inc., San Diego, CA, USA)	>850,000 CpG sites assayed	hg19; annotation performed using IlluminaHumanMethylationEPICanno.ilm10b2.hg19	Not reported; probes removed for detection *p*-value cutoff 0.01, beadcount < 3 in ≥5% of patients, SNP-associated probes with MAF > 0.01 within 10 bp of extension site, and X/Y chromosome probes	ChAMP preprocessing; BMIQ normalization on beta values; beta values transformed to M-values for association analysis	White blood cell composition estimated using minfi estimateCellCounts
[11]	Illumina Infinium MethylationEPIC 850K BeadChip array; scanned using Illumina HiScan	>850,000 CpG sites assayed	hg19; annotation performed using IlluminaHumanMethylationEPICanno.ilm10b2.hg19	Not reported; probes removed for detection *p*-value cutoff 0.01, beadcount < 3 in ≥5% of patients, SNP-associated probes with MAF > 0.01 within 10 bp of extension site, and X/Y chromosome probes	ChAMP preprocessing; BMIQ normalization on beta values; beta values transformed to M-values for association analysis	White blood cell composition estimated using minfi estimateCellCounts
[17]	Illumina Infinium MethylationEPIC 850K BeadChip array; scanned using Illumina HiScan	>850,000 CpG sites assayed	CpG chromosome/base-pair positions reported and loci interpreted using UCSC Genome Browser, Alliance of Genome Resources and GeneCards	Final number of CpGs after QC not reported. DNA methylation preprocessing/QC performed jointly for baseline and week-8 samples using RnBeads 2.0 (R v4.2.2)	RnBeads 2.0 preprocessing/QC pipeline in R; no specific normalization algorithm clearly reported	Houseman method (GSE110554 EPIC-specific reference); 6-cell reference
[26]	Illumina Infinium MethylationEPIC 850K BeadChip array; scanned using Illumina HiScan	>850,000 CpG sites assayed	CpG chromosome/base-pair positions reported and loci interpreted using UCSC Genome Browser, Alliance of Genome Resources and GeneCards	669,674 CpGs included in downstream analyses/pathway analysis after QC	RnBeads 2.0 preprocessing/QC pipeline in R; no specific normalization algorithm clearly reported	Houseman method [28]; GSE110554 EPIC-specific reference); 6-cell reference; estimated neutrophils, monocytes, B lymphocytes, NK cells, CD4+ T cells and CD8+ T cells
[27]	Illumina Infinium MethylationEPIC BeadChip for DNA methylation	>850,000 CpG sites assayed	hg38 coordinates downloaded on 1 November 2025	816,087 autosomal probes were retained for downstream DNAm analysis after QC. For the functional methylation-expression analysis, 316,724 methylation sites annotated to 12,869 expressed leukocyte genes were considered.	Inter-array normalization used preprocessFunnorm, including noob background correction, dye normalization, and correction for the first two principal components of internal control probes. β-values were logit-transformed to M-values for differential methylation analysis.	No reference-based DNAm cell-type deconvolution method was reported.

Abbreviations: BMIQ, Beta-Mixture Quantile; CpG, cytosine-phosphate-guanine dinucleotide; EWAS, epigenome-wide association studies; MAF, minor allele frequency; MDD, major depressive disorder; PBMCs, peripheral blood mononuclear cells; QC, quality control; VIF, variance inflation factor.

**Table 3:** Statistical methods analysis and key EWAS findings in longitudinal studies of treatment response in MDD.

Authors	Statistical Model	Covariates	Multiple Testing Correction	Significant Findings (DMPs/DMRs)	Direction	Effect Size	FDR/q-Value	Pathway Analysis
[14]	Repeated-measures ANOVA/lmer; global DNAm and probe-level DMP analyses tested time, response, and response × time effects	No covariate-adjusted EWAS model reported; responder/non-responder clinical and demographic comparisons performed; X/Y chromosomes excluded	FDR < 5% plus methylation variance threshold > 0.1	13 significant CpG sites across 10 genes/loci: *RNF175*, *RNF213*, *TBC1D14*, *TMC5*, *WSCD1*, *AC018685.2*, *AC098617.1*, *CLCN3P1*; treatment-course CpGs: *AQP10*, *TRERF1*; no DMR analysis	Response-associated CpGs showed stable responder/non-responder differences across time; *AQP10* and *TRERF1* showed treatment-course effects; exact per-locus direction not reported	Δβ, M-value change and CI not reported	FDR < 0.05 for significant CpGs; exact FDR values reported for CpGs in where available	Not reported
[15]	limma mixed linear model with participant ID as blocking factor; tested response, timepoint, and response × timepoint effects; response modeled as binary response and continuous ΔHDRS; baseline methylation prediction models tested	5 estimated white blood cell fractions, 10 control-probe PCs, age, sex, and smoking status; 5th control-probe PC omitted when collinear with sex	CpG-level FDR q < 0.05; suggestive CpG threshold *p* < 1 × 10^−5^; DMRs tested using comb-p and Šídák correction	1 FDR-significant DMP for binary ECT response: *TNKS*. No FDR-significant CpGs for continuous ΔHDRS. 2 significant ΔHDRS-associated DMRs: *BLCAP*, *NNAT* (chr 20), and *LRATD2*/*FAM84B* (chr 8)	Direction of top DMPs/DMRs not reported	Δβ, M-value change and CI not reported	*TNKS* cg10005358: *p* = 7.2 × 10^−8^, FDR = 0.0498. *FKBP5* cg01294490 for continuous ΔHDRS: *p* = 4.5 × 10^−7^, FDR = 0.3106. DMR chr20 *BLCAP*/*NNAT*: Šídák *p* = 4.2 × 10^−5^. DMR chr8 *LRATD2*/*FAM84B*: Šídák *p* = 0.0031	missMethyl v1.12.0 GO analysis on CpGs passing *p* < 1 × 10^−5^; no significant pathways
[12]	limma mixed linear model with patient as blocking factor; DMR analysis with bumphunter using ≥8 probes/cluster, maximum 300 bp between probes and 250 permutations	White blood cell fractions and 2 control-probe PCs; additional models included MADRS symptom variation and response status	DMPs reported at suggestive *p* ≤ 1 × 10^−5^; DMRs considered at adjusted *p* ≤ 0.05 or ≤0.10, with relevant DMRs defined by adjusted *p*-value area ≤ 0.05; Benjamini–Hochberg correction	Whole cohort: 3 nominal T0–T1 DMPs, 4 nominal symptom-variation DMPs and 3 nominal response-status DMPs; none FDR-significant. Whole-cohort DMRs: 54 nominal T0–T1 DMRs, 9 with adjusted *p*-value area ≤ 0.10; 21 nominal symptom-variation DMRs and 26 response DMRs, with 4 response DMRs at adjusted *p*-value area ≤ 0.10; none FDR-significant. Female subgroup: significant DMRs for symptom variation and response status.	Direction of top DMPs/DMRs not reported	Δβ, M-value change and CI not reported	Whole-cohort DMPs/DMRs did not survive FDR correction. Female symptom-variation DMRs: adjusted *p*-value area = 0.0313–0.0448 for *ZFP57*, *LAT*, *DLX4*, *POT1*, *POLD4*. Female response-status DMRs adjusted *p*-value area = 0.0416–0.0417 for *KBTBD11*, *PPP1R14A*, *ARFGAP1*, *RIPOR2*, *FAM30A*, *DLX4*, *LAT*, *SNORD34*, *POLD4*	clusterProfiler enricher/enrichGO using graphite, msigdbr and hsa-ord-db gene sets; pathway enrichment adjusted *p* ≤ 0.2. No relevant enrichment in the female T0–T1 model without covariates. Female symptom-variation models enriched transcriptional activity, growth factor activity, alcoholism pathway, DNA maintenance, MYC targets V2 and transcription-factor targets. Female response-status models enriched oxidative stress, glutamate receptor binding, neuron projection membrane and axolemma terms.
[11]	Clinical efficacy tested with repeated-measures ANOVA; EWAS analysis used limma mixed linear model with patient as blocking factor; DMR analysis used bumphunter	White blood cell fractions and 2 PCs from EPIC control probes; additional models included trauma-focused psychotherapy type, MADRS symptom variation, response at T12, and relapse at T26	DMPs reported at suggestive *p* ≤ 1 × 10^−5^; DMRs considered significant/relevant at adjusted *p*-value area ≤ 0.05; enrichment significance adjusted *p* ≤ 0.1; *p*-values adjusted using Benjamini–Hochberg	Whole cohort T0–T12: 9 nominal DMPs, none significant without covariates. With clinical symptom variation as covariate: 5 FDR-significant DMPs. T0/T8/T12 analysis: 5 FDR-significant DMPs with clinical symptom variation. DMR analysis T0–T12: 110 significant DMRs in whole cohort. EMDR subgroup: 141 significant DMRs. TF-CBT subgroup: 9 nominal DMRs, none significant after correction	Direction of top DMPs/DMRs not reported	Δβ, M-value change and CI not reported	Exact top-locus q-values not reported; DMPs significant at q ≤ 0.05 in symptom-change models; DMRs significant/relevant at adjusted *p*-value area ≤ 0.05	clusterProfiler enricher/enrichGO using graphite, msigdbr and hsa-ord-db gene sets. Whole-cohort DMR genes enriched TNFR2 non-canonical NF-κB pathway, TNF receptor superfamily/non-canonical NF-κB pathway, TNF receptor binding and immune/inflammatory GO terms. EMDR subgroup enriched for inflammatory response, TNF signaling pathway, TNFR2 non-canonical NF-κB and transcription factor target sets
[17]	Cross-sectional EWAS at baseline and week 8 used limma via RnBeads comparing cognitively impaired/non-impaired patients; paired longitudinal analysis in cognitive improvers used Welch’s *t*-test; overlap of top 1% CpGs from week-8 and paired analyses prioritized state/trait signals	Cross-sectional models included sex, age, MADRS score, years of education, height, weight, estimated cell fractions and surrogate variables; 18 surrogate variables at baseline and 17 at week 8. Paired Welch’s *t*-test included no additional confounders.	Genome-wide significance *p* < 5 × 10^−8^; top CpGs reported at *p* < 3 × 10^−5^; exploratory overlap used top 1% CpGs from week-8 and paired longitudinal analyses	No genome-wide significant CpGs at baseline, week 8 or paired longitudinal analysis. Baseline top CpG: cg10962945, *p* = 6.61 × 10^−7^. Week-8 top CpG: cg13620631 in *NTRK2*, *p* = 5.56 × 10^−7^. Paired longitudinal improver analysis identified 65 CpGs overlapping between the top 1% of the paired analysis and week-8 analysis. No DMR analysis reported	Baseline cg10962945 hypomethylated in cognitively impaired patients. Week-8 *NTRK2* cg13620631 and *SLC25A21* cg09278723 hypermethylated in impaired patients; *IL5RA* cg25791578 hypomethylated in impaired patients. Longitudinal *EBF3* cg19659218 increased over time in cognitive improvers.	Mean methylation differences: cg10962945 − 0.376%; *NTRK2* cg13620631 + 0.42%; *SLC25A21* cg09278723 + 2.0%; *IL5RA* cg25791578 − 2.73%; *EBF3* cg19659218 + 1.27%.	No CpGs reached genome-wide significance; FDR/q-values not reported. Reported *p*-values: cg10962945 *p* = 6.61 × 10^−7^; *NTRK2* cg13620631 *p* = 5.56 × 10^−7^; *EBF3* cg19659218 *p* = 6.45 × 10^−5^	No formal pathway enrichment analysis reported. CpGs interpreted using UCSC Genome Browser, Alliance of Genome Resources and GeneCards; highlighted *NTRK2*/*BDNF*, *EBF3* transcriptional regulation, *IL5RA*/immune-inflammatory loci and cognition/depression-related regulatory regions
[26]	Week-8 cross-sectional response vs. non-response analysis used limma/RnBeads; paired longitudinal response, remission and stable-course analyses used Welch’s *t*-test	Cross-sectional model: biological sex, age, years of education, body weight, height, estimated cell fractions and 16 surrogate variables (week 8). Paired longitudinal models include no additional confounders. Baseline follow-up of top CpGs used cell types, demographics and baseline MADRS.	Genome-wide CpG threshold *p* < 5 × 10^−8^; top CpGs reported at *p* < 3 × 10^−5^; top 1% CpGs (*n* = 6696) used for overlap analyses	No genome-wide significant CpGs. Response vs. non-response week 8: 10 CpGs with *p* < 3 × 10^−5^. Paired longitudinal response: 13 CpGs with *p* < 3 × 10^−5^. Paired longitudinal remission: 11 CpGs with *p* < 3 × 10^−5^, including one near genome-wide significance. Stable-course analysis: 14 CpGs with *p* < 3 × 10^−5^. No DMR analysis.	Response vs. non-response: *IQSEC1* cg01601845 and *DRD4* cg02762115 hypomethylated in responders; other top response CpGs showed mixed hyper-/hypomethylation. Longitudinal response: *SOX4* cg22274825 relatively hypermethylated at week 8 vs. baseline. Longitudinal remission: *LIN37* cg02327902 relatively hypermethylated post-intervention.	Mean methylation differences: *IQSEC1* cg01601845 1.14%; *DRD4* cg02762115 3.23%; *SOX4* cg22274825 + 1.93%; *LIN37* cg02327902 + 1.23%; CI NR.	No CpGs reached genome-wide significance. FDR/q-values not reported. Reported *p*-values: *IQSEC1* cg01601845 *p* = 1.53 × 10^−6^; *SOX4* cg22274825 *p* = 5.10 × 10^−6^; *LIN37* cg02327902 *p* = 7.74 × 10^−8^	methylGSA/methylglm GO analysis across 669,674 CpGs; GO pathways reported at *p* < 0.05. Response vs. non-response: 77 GO pathways, e.g., by telomere maintenance and organization. Longitudinal response: 67 GO pathways, e.g., sodium ion transmembrane transporter activity, solute symporter activity. Longitudinal remission: 46 GO terms, e.g., phosphatase regulator activity, protein phosphatase regulator activity, postsynaptic density. Stable-course analysis returned basic cellular-process GO terms.
[27]	limma v3.50.0 tested DNAm change from T0 to T8 against HAM-D T8/T0 ratio, with batch and subject fixed effects; secondary limma mixed model used duplicateCorrelation. RNA-seq differential expression used DESeq2. DNAm-expression links used Spearman correlation	Primary DNAm model: batch and subject. Secondary DNAm model: sex, batch, age bins and first four PCs, with subject as blocking factor. RNA-seq model: sex, age, RIN and batch. DNAm-expression correlations adjusted methylation for batch, age and sex, and RNA-seq for age, sex and RIN. Blood-cell counts were assessed but no reference-based DNAm cell-deconvolution model was reported.	CpG-level FDR correction; GO *p*-values FDR-adjusted. Functional DNAm-expression analysis used stepwise exploratory thresholds: DNAm *p* ≤ 0.01, T8 expression FDR ≤ 0.2 and DNAm-expression Spearman *p* ≤ 0.05.	One FDR-significant DMP: cg12958009/*SCN7A*. Second-ranked cg17944171/*IQANK1*-*FAM83H-AS1* was nominal. No DMR analysis. DNAm-expression integration identified 10 genes: *ABCB10*, *ATP11A*, *CAMSAP1*, *GOLGA3*, *MGAT5*, *NSD1*, *RALGAPA2*, *RUNX1*, *SNTB2* and *VPS13B*.	*SCN7A* methylation decreased with greater clinical improvement; *IQANK1*/*FAM83H*-*AS1* methylation increased with greater improvement. Functional DNAm sites showed mixed direction: *ATP11A* and *GOLGA3* increased methylation with improvement, whereas *ABCB10*, *CAMSAP1*, *MGAT5*, *NSD1*, *RALGAPA2*, *RUNX1*, *SNTB2* and *VPS13B* decreased. All 10 genes showed higher T8 expression with greater improvement.	Δβ/CI not reported for top CpGs. Functional DNAm logFC range −0.25 to 0.35: *ABCB10* 0.19, *ATP11A* −0.21, *CAMSAP1* 0.35, *GOLGA3* −0.25, *MGAT5* 0.24, *NSD1* 0.23, *RALGAPA2* 0.34, *RUNX1* 0.20, *SNTB2* 0.14 and *VPS13B* 0.25.	*SCN7A* cg12958009 FDR *p* = 0.04; *IQANK1*/*FAM83H*-*AS1* cg17944171 FDR *p* = 0.15. Secondary model: *SCN7A p* = 1.5 × 10^−6^ and *IQANK1 p* = 3.5 × 10^−6^, not FDR-significant. Functional DNAm *p* = 0.0007–0.0095.	clusterProfiler GO analysis using top 0.1% probesets annotated to genes. Small GTPase-mediated signaling enriched in whole-sample and expressed-gene analyses; Wnt signaling was the top pathway in sex-stratified analyses. No DMR-based pathway analysis.

Abbreviations: CI, confidence interval; CpG, cytosine-phosphate-guanine dinucleotide; DMP, differentially methylated probes; DMR, differentially methylated regions; ECT, electroconvulsive therapy; EMDR, Eye Movement Desensitization and Reprocessing; EWAS, epigenome-wide association studies; FDR, false discovery rate; GO, gene ontology; HDRS, Hamilton Depression Rating Scale; MADRS: Montgomery–Åsberg Depression Rating Scale; MDD, major depressive disorder; PC, principal component; RIN, RNA integrity number; TF-CBT, trauma-focused cognitive behavior therapy.

## Data Availability

No new data were created or analyzed in this study. Data sharing is not applicable to this article.
